# A mitophagy-related gene signature associated with prognosis and immune microenvironment in colorectal cancer

**DOI:** 10.1038/s41598-022-23463-8

**Published:** 2022-11-04

**Authors:** Cong Zhang, Cailing Zeng, Shaoquan Xiong, Zewei Zhao, Guoyu Wu

**Affiliations:** 1 Hospital of Chengdu University of Traditional Chinese Medicine, Chengdu, 610072 Sichuan China; 2 Chengdu University of Traditional Chinese Medicine, Chengdu, 610072 Sichuan China

**Keywords:** Cancer genomics, Cancer, Molecular biology

## Abstract

Colorectal cancer (CRC) is a heterogeneous disease and one of the most prevalent malignancies worldwide. Previous research has demonstrated that mitophagy is crucial to developing colorectal cancer. This study aims to examine the association between mitophagy-related genes and the prognosis of CRC patients. Gene expression profiles and clinical information of CRC patients were obtained from The Cancer Genome Atlas (TCGA) and Gene Expression Omnibus (GEO) databases. Univariate Cox regression and the least absolute shrinkage and selection operator (LASSO) regression analysis were applied to establish a prognostic signature using mitophagy related genes. Kaplan–Meier and receiver operating characteristic (ROC) curves were used to analyze patient survival and predictive accuracy. Meanwhile, we also used the Genomics of Drug Sensitivity in Cancer (GDSC) database and Tumor Immune Dysfunction and Exclusion (TIDE) algorithm to estimate the sensitivity of chemotherapy, targeted therapy and immunotherapy. ATG14 overexpression plasmid was used to regulate the ATG14 expression level in HCT116 and SW480 cell lines, and cell counting kit-8, colony formation and transwell migration assay were performed to validate the function of ATG14 in CRC cells. A total of 22 mitophagy-driven genes connected with CRC survival were identified, and then a novel prognostic signature was established based on 10 of them (*AMBRA1*, *ATG14*, *MAP1LC3A*, *MAP1LC3B*, *OPTN*, *VDAC1*, *ATG5*, *CSNK2A2*, *MFN1*, *TOMM22*). Patients were divided into high-risk and low-risk groups based on the median risk score, and the survival of patients in the high-risk group was significantly shorter in both the training cohort and two independent cohorts. ROC curve showed that the area under the curves (AUC) of 1-, 3- and 5-year survival were 0.66, 0.66 and 0.64, respectively. Multivariate Cox regression analysis confirmed the independent prognostic value of the signature. Then we constructed a Nomogram combining the risk score, age and M stage, which had a concordance index of survival prediction of 0.77 (95% CI 0.71–0.83) and more robust predictive accuracy. Results showed that CD8+ T cells, regulatory T cells and activated NK cells were significantly more enriched in the high-risk group. Furthermore, patients in the high-risk group are more sensitive to targeted therapy or chemotherapy, including bosutinib, elesclomol, lenalidomide, midostaurin, pazopanib and sunitinib, while the low-risk group is more likely to benefit from immunotherapy. Finally, in vitro study confirmed the oncogenic significance of ATG14 in both HCT116 and SW480 cells, whose overexpression increased CRC cell proliferation, colony formation, and migration. In conclusion, we developed a novel mitophagy-related gene signature that can be utilized not only as an independent predictive biomarker but also as a tool for tailoring personalizing treatment for CRC patients, and we confirmed ATG14 as a novel oncogene in CRC.

## Introduction

Colorectal cancer (CRC) is the third most common malignancy worldwide and is considered the second leading cause of cancer-related death, causing an estimated more than 800,000 deaths in 2018^1^. It is a heterogeneous disease that develops as a result of the accumulation of mutations brought on by a combination of environmental, genetic, and other risk factors over time^2^. The main strategy for treating unresectable metastatic CRC is systemic therapy, including cytotoxic chemotherapy, biological therapy such as EGFR antibody, chemotherapy, immunotherapy and combination therapy. Among newly diagnosed colorectal cancer patients, 20% of them presented with metastatic disease, and another 25% with localized disease will develop metastases later^3^. Although timely treatment can be initiated based on early detection results, the prognosis of patients with metastatic CRC (mCRC) remains poor, with a 5-year survival rate of only 12.5% for CRC patients in the United States^4,5^. Therefore, disease metastasis and recurrence seriously affect CRC patients’ prognosis^6^. Even though immune checkpoint inhibitors have been reported to have achieved significant efficacy in a cohort of CRC patients^7–9^, however, they remain ineffective in mismatch repair-proficient (pMMR) patients, including those with microsatellite stable (MSS) or microsatellite instability-low (MSI-L)^10^. Therefore, it is of clinical importance to determine CRC patients’ prognosis and which patients will benefit from immunotherapy.

Mitophagy, a selective autophagy process, is a fundamental mechanism controls the quality and quantity of mitochondria by degrading dysfunctional mitochondria, conserved from yeast to humans^11,12^. It plays a crucial role in the survival of cancer cells by influencing the metabolic reprogramming of mitochondria in tumors and the accumulation/elimination rate of damaged mitochondria through different mechanisms to maintain mitochondrial homeostasis^12^. On the other hand, previous studies have identified mutations or downregulation in the expression of some mitophagy adaptors in certain kinds of cancer, thus demonstrating the suppressive role for mitophagy on tumors^13^. Recently, Chen et al. found that DJ-1 (Parkinson’s disease-associated protein 7, PARK7) activated mitophagy can remove dysfunctional mitochondria and inhibit the apoptosis of metastatic colorectal adenocarcinoma cells, thereby promoting the progression of CRC^14^. Additionally, prior research has shown that Tanshinone IIA (Tan IIA) could modulate mitochondrial homeostasis by altering mitophagy, which has suppressive effects on CRC^15^. Although previous research had revealed a link between genes associated with mitophagy and the prognosis in several cancer types, it had mostly concentrated on the function of a single gene.

In this study, we comprehensively assessed the prognostic value of mitophagy-related genes in CRC based on the expression profiles of the TCGA-CRC, GSE17536 and GSE24551 datasets. We finally screened 10 mitophagy-related genes by Least absolute shrinkage and selection operator (LASSO) regression analysis, and built a risk model based on them to predict the prognosis of CRC. We also analyzed the clinical characteristics, gene mutation profiles, tumor immune microenvironment, and drug sensitivity of CRC patients with different risk levels, and the results showed that the two risk groups of patients showed significantly different characteristics in these aspects. In conclusion, this model is helpful in predicting the prognosis of CRC patients and provide a reference for clinical chemotherapy and immunotherapy.

## Materials and methods

### Data curation

RNA sequencing (RNA-seq), clinicopathological information, and somatic mutation data of 354 CRC patients were retrieved and downloaded from The Cancer Genome Atlas program (TCGA, https://portal.gdc.cancer.gov/) through cBioPortal for Cancer Genomics (https://cbioportal.org/). Meantime, RNA-seq and clinicopathological information of 337 CRC patients (GSE17536 and GSE24551) were downloaded from the Gene Expression Omnibus (GEO, https://www.ncbi.nlm.nih.gov/geo/) database as two independent external validation cohorts. Forty-six mitophagy-related genes were obtained from the GO (http://geneontology.org/) and KEGG (https://www.genome.jp/kegg/) database.

### Construction and validation of the prognostic gene signature

Univariate Cox proportional hazard regression analysis was used to screen mitophagy-related genes associated with overall survival in the TCGA cohort (p < 0.1). To (indirectly) reduce the risk of a Type II error ("false negative"), we set the significance level as 0.1. The least absolute shrinkage and selection operator (LASSO) Cox regression model was performed to find the best gene signature from the genes obtained in univariate analysis by using the “glmnet” R package. The coefficient and expression of mitophagy related genes in the risk model were obtained, and the risk score of each patient was calculated. The formula is as follows: risk score = ${\sum }_{j=1}^{n}{Expr}_{genej}*{Coef}_{genej}$, with $Expr$ indicating the level of gene expression and $Coef$ representing the coefficient of gene. The median risk score was selected as the cutoff value to divide TCGA-CRC patients into high-risk group and low-risk group. Survival analysis between groups was conducted based on the overall survival data, and p < 0.05 was considered as statistically significant. Kaplan–Meier survival curves were plotted by the “survival” and “survminer” packages. To verify the accuracy and validity of signatures, “timeROC” R package was used to calculate the area under the curve (AUC) values at 1-, 3-, and 5-years derived from receiver operating characteristic (ROC) analysis. Prognostic gene signature was validated in two independent cohorts from GEO (GSE17536 and GSE24551). Univariate and multivariate Cox regression analyses were performed using clinical parameters and risk scores to assess the independent prognostic value of the signature.

### Enriched pathway analysis

To analyze the potential functions of differential expressed genes of mitophagy-related signature, “clusterProfiler” R package was used for functional annotation of Gene Ontology (GO) and pathway enrichment analysis of Kyoto Encyclopedia of Genes and Genomes (KEGG)^16–18^. GO enrichment was carried out at three levels: cellular component (CC), biological process (BP) and molecular function (MF). To reveal biological process in the high-risk and low-risk groups, Gene Set Enrichment Analyses (GSEA) was performed by “ClusterProfiler” package in R studio. The false discovery rate (FDR) q < 0.01 was considered statistically significant.

### Development of a nomogram

Then, a Nomogram prognostic prediction model was constructed based on risk scores, age, and M stage using the “rms” R package. AUC of the ROC curve, Harrell's concordance index (C-index), and calibration plots to compare predicted and observed overall survival were used to assess the prognostic performance of the established Nomogram. The closer the C-index is to 1, the higher the accuracy of the results predicted by the model^19^.

### Evaluation of infiltrating immune cells

CIBERSORT algorithm was used to calculate the tumor-infiltrating immune cells^20^. The algorithm is built on normalized gene expression data and a gene signature matrix (LM22) that annotates 22 different subtypes of immune cells, which data could be downloaded from The CIBERSORT web portal (https://cibersort.stanford.edu/). When p < 0.05, the immune cell infiltration abundance was considered statistically different between tumor samples from the two risk groups.

### Mutation analysis

To explore somatic mutations in CRC patients between low- and high-risk group, we obtained the mutation annotation format (MAF) for TCGA-CRC from the TCGA database, and used the “maftools” R package for analysis and visualization^21^.

### Prediction of response to immunotherapy, chemotherapy, targeted therapy

Using the Genomics of Drug Sensitivity in Cancer (GDSC, https://www.cancerrxgene.org/) database to estimate the sensitivity of each patient to chemotherapy drugs. Screening method for candidate small molecule drugs referred to previously published article^22^. The IC_50_ was quantified via “pRRophetic” R package. Tumor Immune Dysfunction and Exclusion (TIDE) algorithm (http://tide.dfci.harvard.edu/login/)^23^ was used to evaluate the responses of ICB therapies in two groups.

### In vitro analysis of the function of ATG14 in CRC

#### ATG14 overexpression

To regulate the expression level of ATG14 in CRC cells (HCT116 and SW480), pCMV plasmid was transfected using Lipofect8000 (Thermo Fisher, MA, USA) to induce gene overexpression. pCMV-ATG14-GFP vectors and cDNAs for human ATG14 were obtained from COBIOER (Nanjing, China) and Sino Biological Inc (Beijing, China), respectively. Then, the expression level of ATG14 in normal and ATG-overexpressed HCT116 and SW480 cell lines were determined by western blotting. Anti-ATG14 primary antibody (#5504) and anti-β-actin primary antibody (#3700) were obtained from Cell Signaling Technology Co. (MA, USA).

#### Cell proliferation assay

Cell Counting Kit-8 was utilized to measure cellular proliferation (CCK8, Solarbio, China). In a 96-well plate, 2 × 10^3^ vector- or ATG14-overexpressed HCT116 and SW480 cells were planted per well. After 0, 24, 48, and 72 h of culture, samples were incubated with a 10% CCK-8 solution for three hours. Microplate reader was used to measure absorbance at 450 nm (Thermo Fisher, MA, USA).

#### Colony formation and transwell assay

Vector or ATG14 overexpressed HCT116 and SW480 cells were patented in a 6-well plate (5 × 10^2^ cells/well, Corning, USA) containing Roswell Park Memorial Institute 1640 complete culture medium. After 10 days, colonies (> 50 cells) were stained with crystal violet, and counted under a low magnification microscope (Leica, Germany). 5 × 10^4^ vector and ATG14 overexpressed cells were seeded in a transwell chamber (8 μm, Thermo Fisher, MA, USA) containing 300 μl culture medium (with 10% fetal bovine serum, FBS). Then, 1 ml FBS-free culture medium was added to a 24-well plate and after 24 h, the chamber was fixed with paraformaldehyde and stained with crystal violet. Migrating cell numbers were counted under the microscope (Leica, Germany).

### Statistical analysis

Data were processed, analyzed and presented using the R software (v.4.0.3) and related software packages. Kaplan–Meier curves were used to describe the relationship between patient survival time and survival probability. We visualized the risk-related information through charts, including risk score distribution, risk-related survival status, heat maps of prognostic genes, etc. The ROC analysis was used to analyze the sensitivity and specificity of survival prediction using the gene signature risk score, and using AUC as an indicator of prognostic accuracy. Univariate and multivariate Cox regression analysis were used to verify the independence of signatures. In addition, we assessed the correlation between the signature and clinical parameters. p < 0.05 was considered statistically significant.

## Results

### Construction of the prognostic signature based on mitophagy-related genes

**Figure 1:**
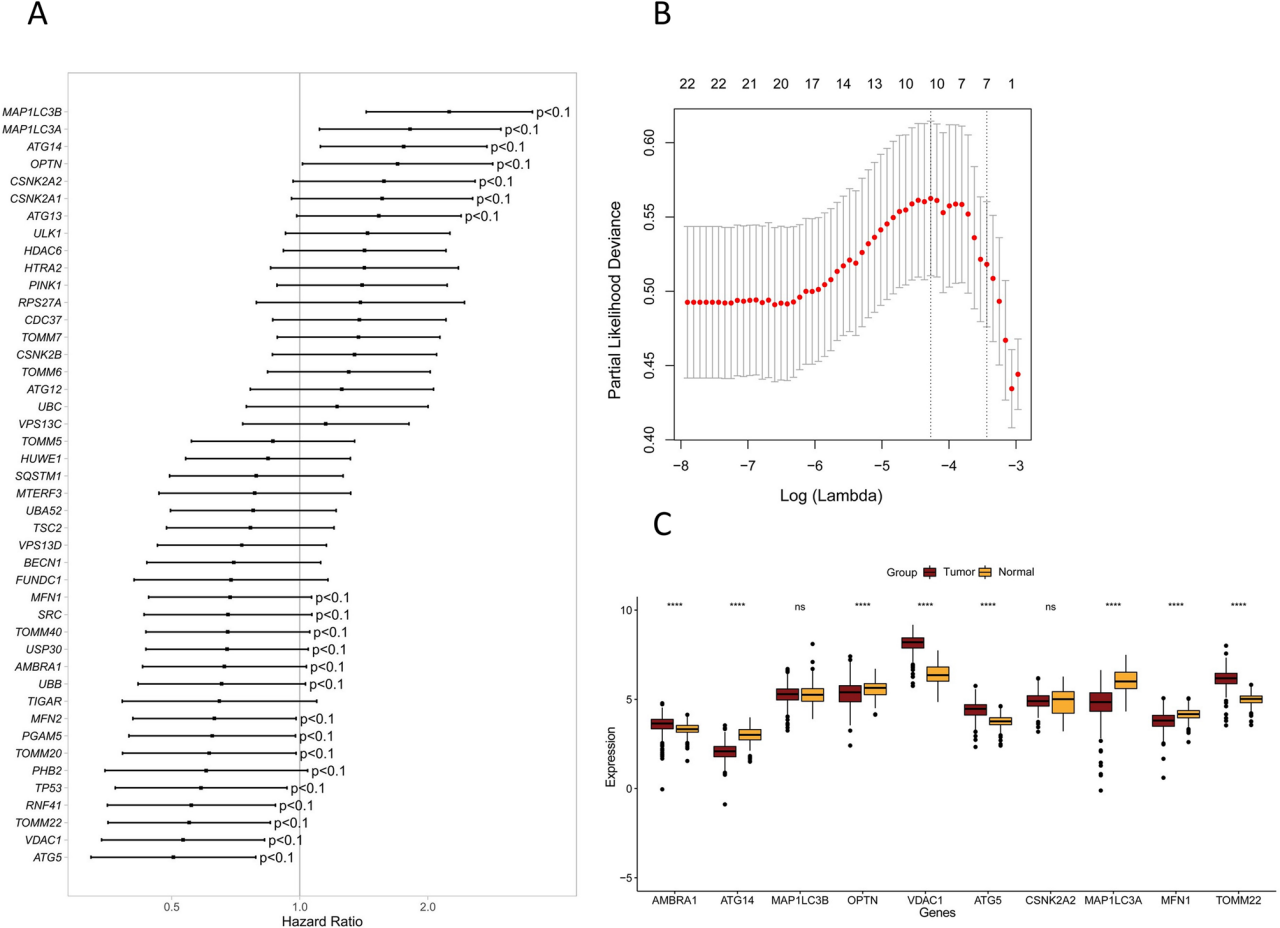
Establishment of the prognostic gene signature based on 10-mitophagy-related genes. (**A**) The mitophagy related genes associated with CRC survival were selected by univariate Cox regression analysis. (**B**) Cross-validation for tuning parameter (lambda) screening in the least absolute shrinkage and selection operator (LASSO) regression model based on minimum criteria for OS. (**C**) Differential expression of the 10 selected genes between normal and CRC tissues.

The flow of this study is shown in Supplementary Fig. 1. In order to identify prognostic mitophagy related genes, we performed univariate Cox regression analysis on these genes, and a total of 22 genes were identified have correlation with the OS of TCGA-CRC (Fig. 1A). Then, LASSO Cox regression analysis was applied, and 10 most prognostic genes (*AMBRA1*, *ATG14*, *MAP1LC3A*, *MAP1LC3B*, *OPTN*, *VDAC1*, *ATG5*, *CSNK2A2*, *MFN1*, *TOMM22*) associated with mitophagy were screened out (Fig. 1B). Notably, the expression of ATG14, OPTN, MAP1LC3A, and MFN1 was significantly downregulated in tumors compared to normal controls, but the expression of AMBRA1, VDAC1, ATG5, and TOMM22 was significantly upregulated (p < 0.0001, Fig. 1C). The risk score was calculated according to their expression levels and Cox regression coefficients and the formula for calculation is as follows: risk score = expression level of *AMBRA1* * (− 0.0666731) + expression level of *ATG14* * 0.26819842 + expression level of *MAP1LC3B* * 0.03411786 + expression level of *OPTN* * 0.26292307 + expression level of *VDAC1* * (− 0.16993759) + expression level of *ATG5* * (− 0.31642485) + expression level of *CSNK2A2* * 0.07033702 + expression level of *MAP1LC3A* * 0.09318544 + expression level of *MFN1* * (− 0.27707284) + expression level of *TOMM22* * (-0.02760961). The characteristics of these genes were shown in Table 1.

**Table 1:** General characteristics of genes in the prognostic gene signature.

Gene symbol	Full name	Hazard ratio	p value	Risk coefficient
AMBRA1	Autophagy and beclin 1 regulator 1	0.665151	0.071512	− 0.0666731
ATG14	Autophagy related 14	1.753384	0.014580	0.26819842
MAP1LC3A	Microtubule associated protein 1 light chain 3 alpha	1.817103	0.017146	0.09318544
MAP1LC3B	Microtubule associated protein 1 light chain 3 beta	2.245661	0.000424	0.03411786
OPTN	Optineurin	1.698439	0.043684	0.26292307
VDAC1	Voltage dependent anion channel 1	0.531979	0.005017	− 0.16993759
ATG5	Autophagy related 5	0.504291	0.002621	− 0.31642485
CSNK2A2	Casein kinase 2 alpha 2	1.577459	0.069813	0.07033702
MFN1	Mitofusin 1	0.685683	0.093788	− 0.27707284
TOMM22	Translocase of outer mitochondrial membrane 22	0.549266	0.007467	− 0.02760961

**Figure 2:**
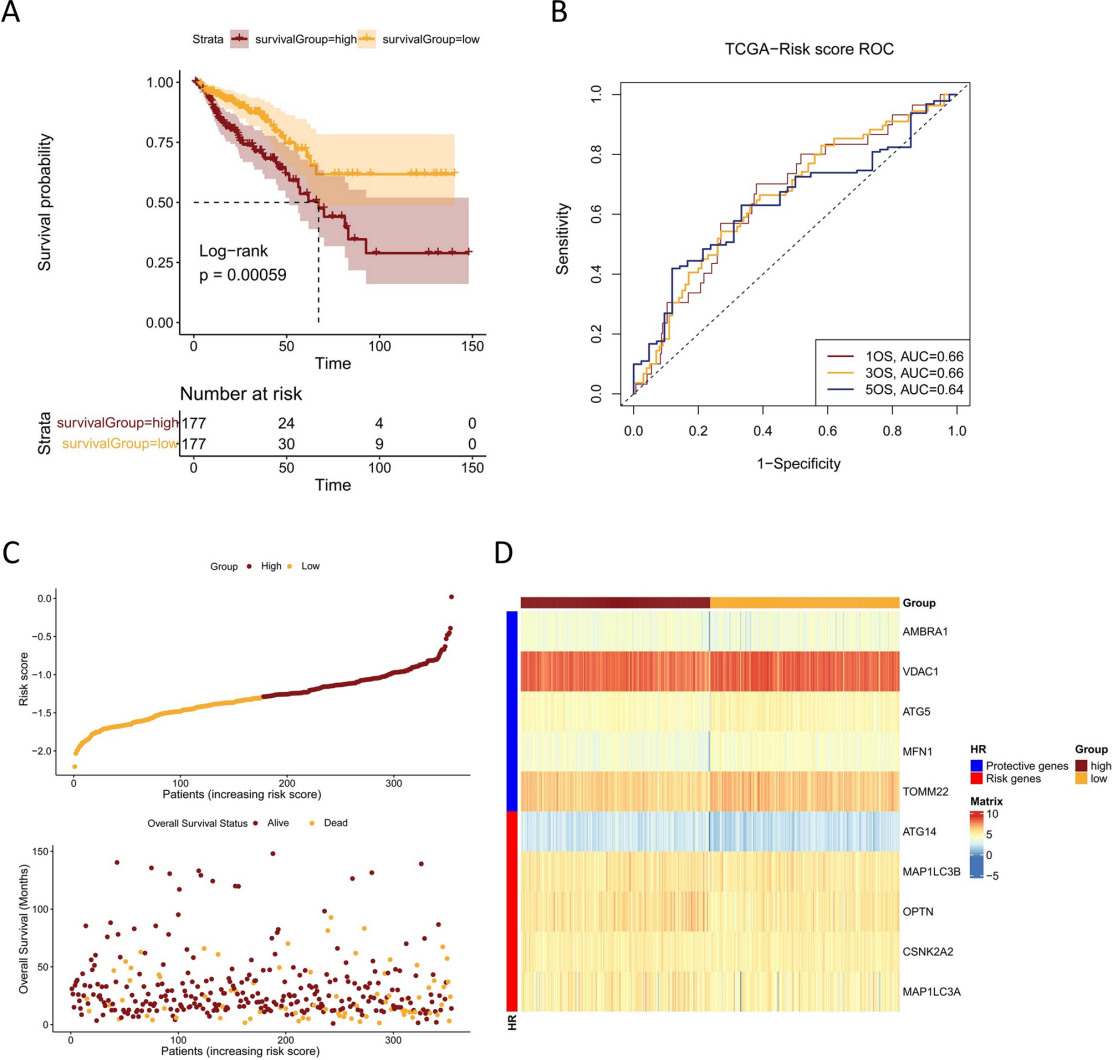
The prognostic value of the signature in the TCGA cohort. (**A**) Kaplan–Meier survival analysis for TCGA-CRC patients with high and low risk scores. (**B**) Time dependent ROC curve analysis of the risk score in TCGA cohort. (**C**) Risk score distribution and survival status for TCGA-CRC patients in high-risk and low-risk groups. (**D**) Heatmap of the mitophagy related genes expression profile for TCGA-CRC patients in high-risk and low-risk groups.

In order to categorize patients from the TCGA-CRC cohort into high and low risk groups, the median risk score was used as the threshold value. To distinguish prognostic differences between the high and low risk groups, a Kaplan–Meier curve based on a log-rank test was applied. The result demonstrated a statistically significant difference in OS between the two TCGA-CRC cohort groups, with patients in the high-risk group having a worse prognosis than that of patients in the low-risk group (median OS 67.3 months vs. not reached, p = 0.00059) (Fig. 2A). Then time-dependent ROC curve was used to verify the accuracy of the mitophagy gene signature and showed that the AUC of 1-, 3- and 5-year survival were 0.66, 0.66 and 0.64, respectively (Fig. 2B), indicating that the mitophagy signature possessed a reliable ability for predicting the prognosis of CRC patients. We then analyzed the relationship between risk score distribution and survival status of CRC patients in the TCGA cohort (Fig. 2C). Patient survival time reduced and the rate of survival rose as the patient's risk score increased. The heatmap showed the expression profile of mitophagy related genes in high-risk and low-risk groups (Fig. 2D). Genes with HR > 1 was regarded as risk genes (ATG14, MAP1LC3B, OPTN, CSNK2A2, MAP1LC3A), whereas those with HR < 1 were considered as protective genes (AMBRA1, VDAC1, ATG5, MFN1, TOMM22). Samples from the high-risk group tended to overexpress risk genes; Patients in the low-risk category, however, have higher levels of protective gene expression.

### Validation of the prognostic gene signature in the independent CRC cohorts

**Figure 3:**
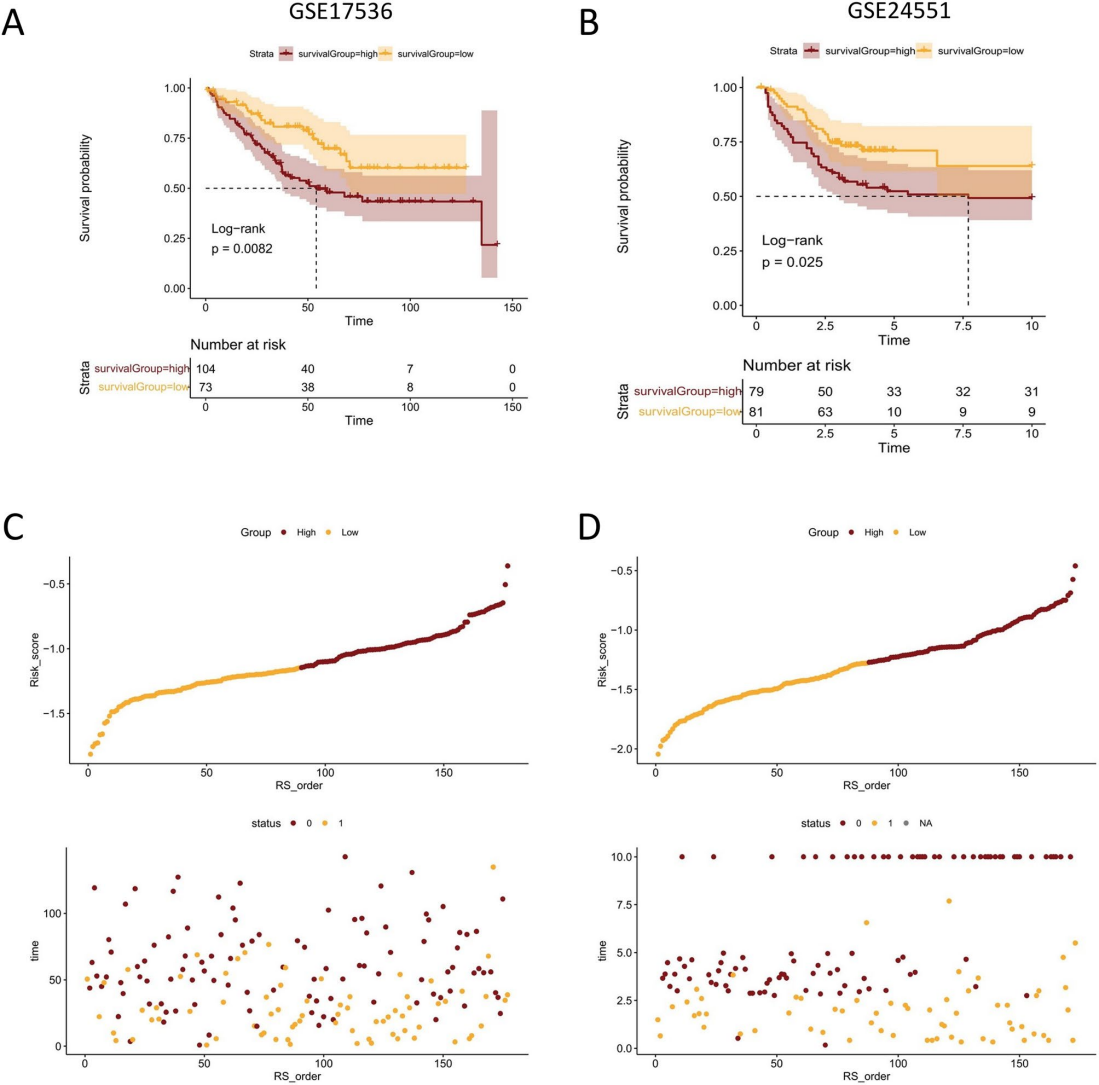
Validation of the prognostic signature in GSE17536 and GSE24551 cohort. (**A**, **B**) Kaplan–Meier survival analysis for GEO-CRC patients with high and low risk scores. (**C**, **D**) Risk score distribution and survival status for GEO-CRC patients in high-risk and low-risk groups.

To validate the accuracy of this signature, we next evaluated the predictive ability of the mitophagy-related gene signature in different CRC cohorts (GSE17536 and GSE24551) from the GEO database. Similarly, the median risk score was used as a cutoff to classify patients in each dataset into high-risk and low-risk groups, respectively. Kaplan–Meier curves based on the log-rank test showed that the high-risk group had a worse prognosis than the low-risk group in both datasets (GSE17536, median OS 54.0 months vs. not reached, p = 0.0082; GSE24551, median OS 7.7 months vs. not reached, p = 0.025; Fig. 3A,B). The relationship between the distribution of risk score and survival status for the two GEO-CRC cohorts were shown in Fig. 3C,D, which were similar to the results of TCGA-CRC cohort. In general, the mitophagy gene signature could stably and accurately predict the prognosis of CRC patients.

### Independent prognostic value of the prognostic gene signature

Subsequently, we assessed the independent prognostic value of mitophagy gene signature. Univariate and multivariate Cox regression analyses were performed to evaluate whether signature-generated risk scores were independent of other clinical parameters (age, MSIsensor score, T, N, M stage) as prognostic factors for CRC patients. Univariate Cox regression analysis showed that in TCGA-CRC cohort, risk score, age, T, N, and M stage were significantly correlated with OS (Table 2). Multivariate Cox regression analysis showed that risk score was independently associated with OS in TCGA-CRC cohort (HR = 2.77, 95% CI 1.27–6.04, p < 0.05) (Table 2). These results further confirmed the high predictive accuracy of mitophagy gene signature, suggesting that the signature could be independently used to predict the prognosis of CRC patients.

**Table 2:** Univariate and multivariate Cox regression analysis of overall survival in TCGA.

	**UniCox**		**MultiCox**	
	**Hazard ratio (95% CI)**	**p value**	**Hazard ratio (95% CI)**	**p value**
Age	2.58 (1.59–4.19)	1.23E − 04	3.70 (2.05–6.68)	1.41E − 05
T stage	2.21 (1.02–4.82)	4.52E − 02	1.59 (0.55–4.56)	3.90E − 01
N stage	2.44 (1.55–3.85)	1.18E − 04	1.78 (0.98–3.23)	5.65E − 02
M stage	3.38 (1.98–5.78)	8.75E − 06	3.54 (1.87–6.70)	1.00E − 04
MSIsensor score	1.00 (0.98–1.02)	8.57E − 01	1.00 (0.97–1.02)	9.08E − 01
Risk score	4.51 (2.32–8.75)	8.44E − 06	2.77 (1.27–6.04)	1.05E − 02

### Relationships between the prognostic gene signature and clinical features

**Figure 4:**
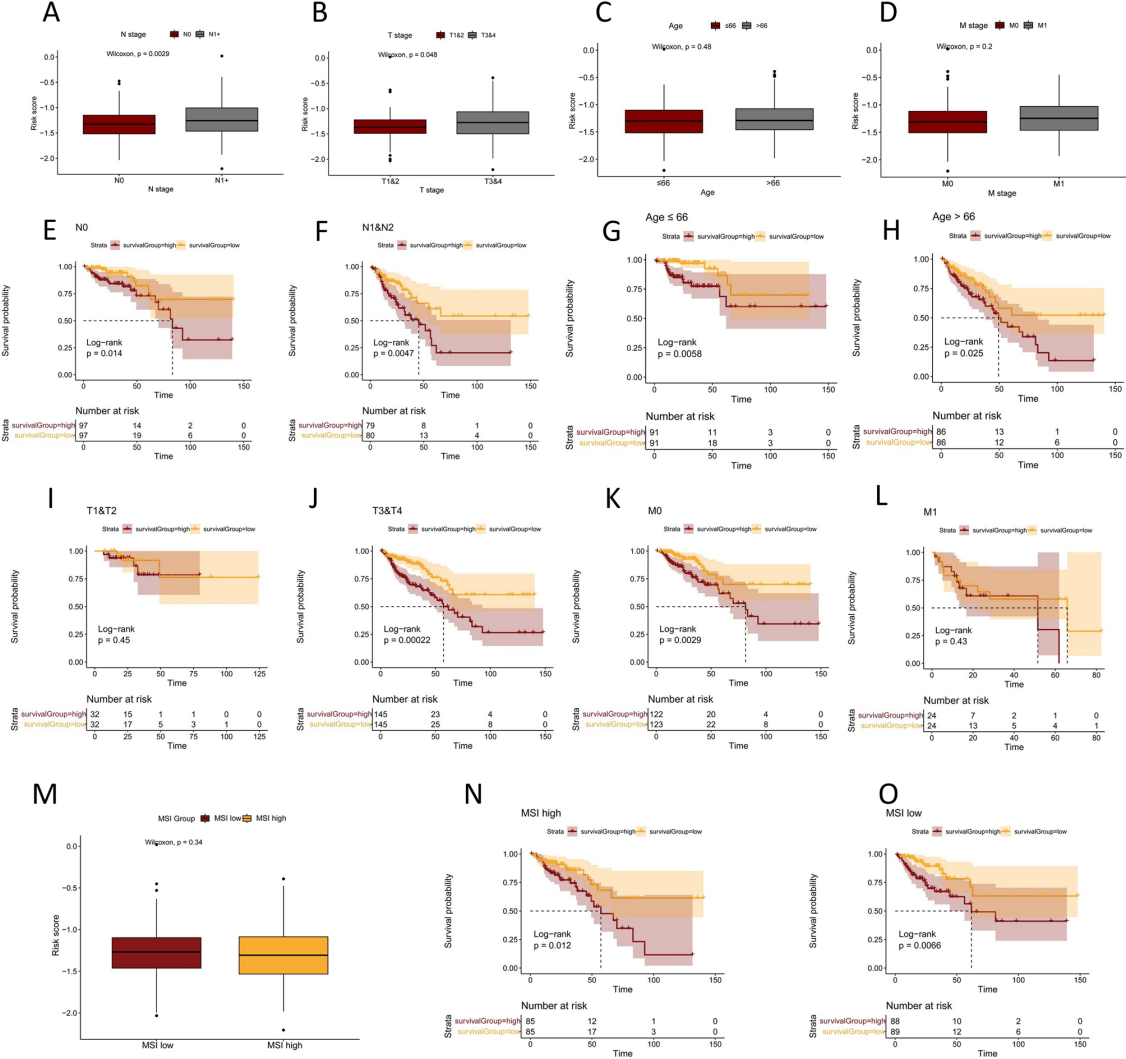
Association between pathologic characteristics and the prognostic signature in the TCGA cohort. (**A**–**D**, **M**) Distribution of the risk score in TCGA-CRC patients stratified by N stage, T stage, age, M stage and MSI. (**E**–**L**, **N**, **O**) The high-risk group showed a poor prognosis than the low-risk group in different clinical stratification.

The CRC cohort from TCGA include a total of 354 patients whose demographic and clinical features were listed in Supplementary Table 1. We further analyzed the relationship between the gene signature and clinical parameters of the TCGA-CRC patients. The results showed that the risk scores of patients with positive lymph node metastasis (N1&2) were significantly higher than those without lymph node metastasis (p = 0.0029, Fig. 4A). Similar results were also observed in T stage (p = 0.048, Fig. 4B). However, the risk score was not significantly associated with age and M stage (Fig. 4C,D). Based on these clinical features, patients were stratified with different clinical feature to verify the effectiveness of the prognostic signature. The results showed that the signature could be well applied to each subgroup of N stage (Fig. 4E,F), age (Fig. 4G,H). However, under T and M stage stratification, the signature was only effective for patients with T 3&4 and or M0 stage (Fig. 4I–L), which may be attributed by limited the sample size. Additionally, in each stratum of these clinical characteristics, the OS of patients in the high-risk group was lower than that of patients in the low-risk group. In order to further explore the relationship between gene signature and microsatellite status in TCGA-CRC patients, we analyzed the correlation between risk score and microsatellite instability status in 347 CRC patients with this data, and the results showed that there was no difference in the risk score between MSI and non- MSI group (Fig. 4M). Meanwhile, regardless of whether patients were MSI or not, high-risk group showed significantly worse prognosis than the low-risk group (Fig. 4N,O). These results suggested that mitophagy related gene signature remained an important prognostic factor when stratifying CRC patients according to different clinical parameters.

### Enrichment analysis of the mitophagy signature

**Figure 5:**
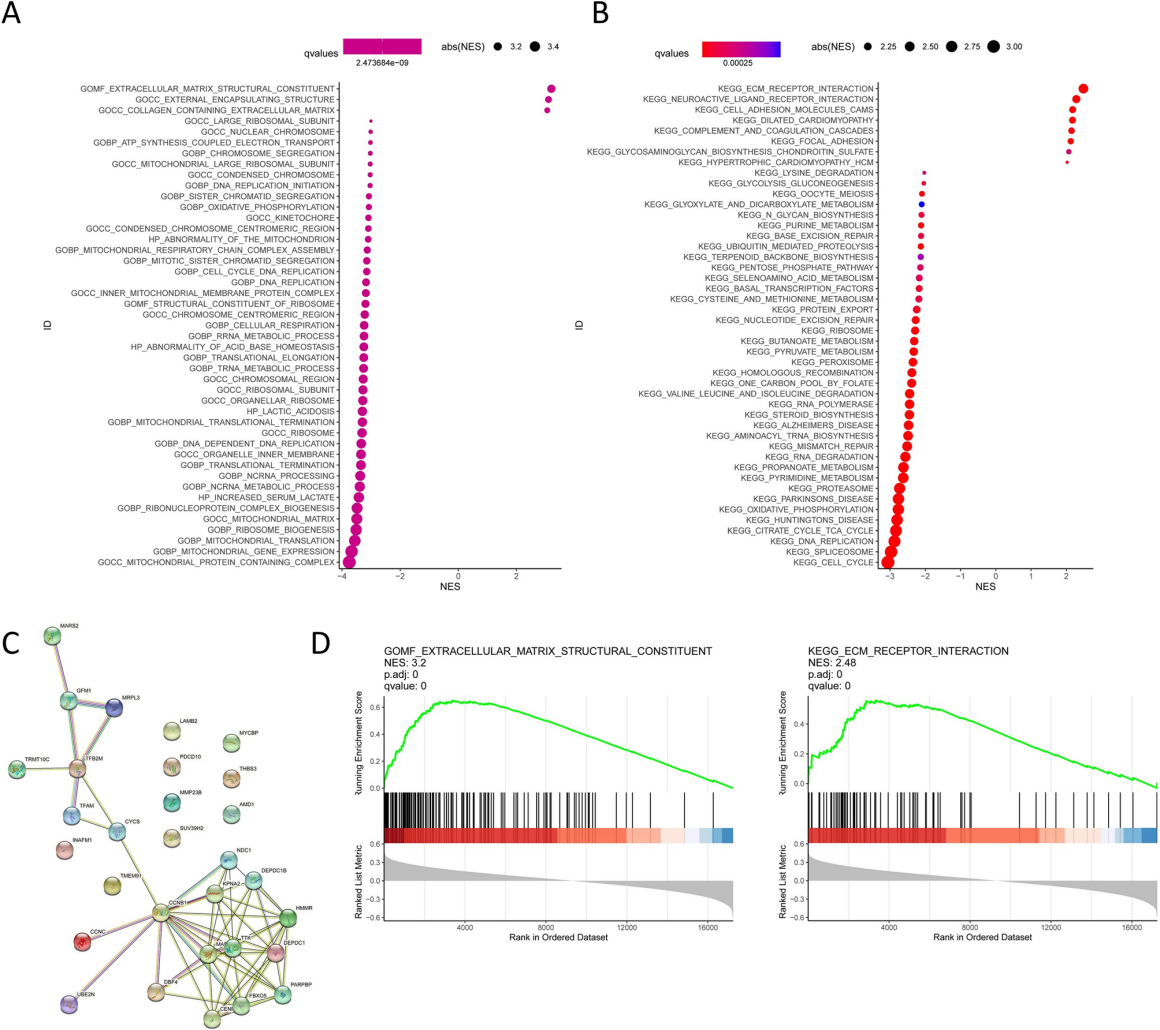
GO, KEGG, protein–protein interaction (PPI) network and GSEA analysis. (**A**) GO and (**B**) KEGG analysis of 10 mitophagy related genes. (**C**) PPI network of mitophagy related genes, revealing their intrinsic connections. (**D**) GSEA analysis of the mitophagy related genes between high and low risk groups.

We performed enrichment analysis of differentially expressed genes (DEGs) in the high-risk and low-risk groups of the TCGA-CRC cohort. DEGs between high-risk and low-risk groups were determined by the cutoff log_2_|FC| > 1 and FDR < 0.05, and annotated GO enrichment analysis and KEGG pathway analysis were then performed. GO analysis showed that the enrichment results of DEGs mainly involved GOBP and GOCC (Fig. 5A). First, for GOBP, DEGs were significantly enriched in biological processes such as chromosome segregation, DNA replication; for GOCC, DEGs were significantly enriched in chromosomal regions, condensed chromosomes, centromeres, ribosomes, etc. KEGG analysis showed that DEGs were significantly enriched in pathways such as cell cycle, DNA replication, and Parkinson's disease (Fig. 5B). In addition, we further performed protein–protein interaction network analysis on the DEGs (Fig. 5C). We found that DEGs exhibited close associations, among which the centrally located CCNB1 and KPNA2 may have potential roles in tumor progression. GSEA analysis was performed based on high-risk and low-risk groups. As shown in Fig. 5D, the high-risk group was significantly correlated with extracellular matrix structural constituent (NES = 3.2) and ECM receptor interaction (NES = 2.48).

### Construction and validation of the prognostic prediction Nomogram

**Figure 6:**
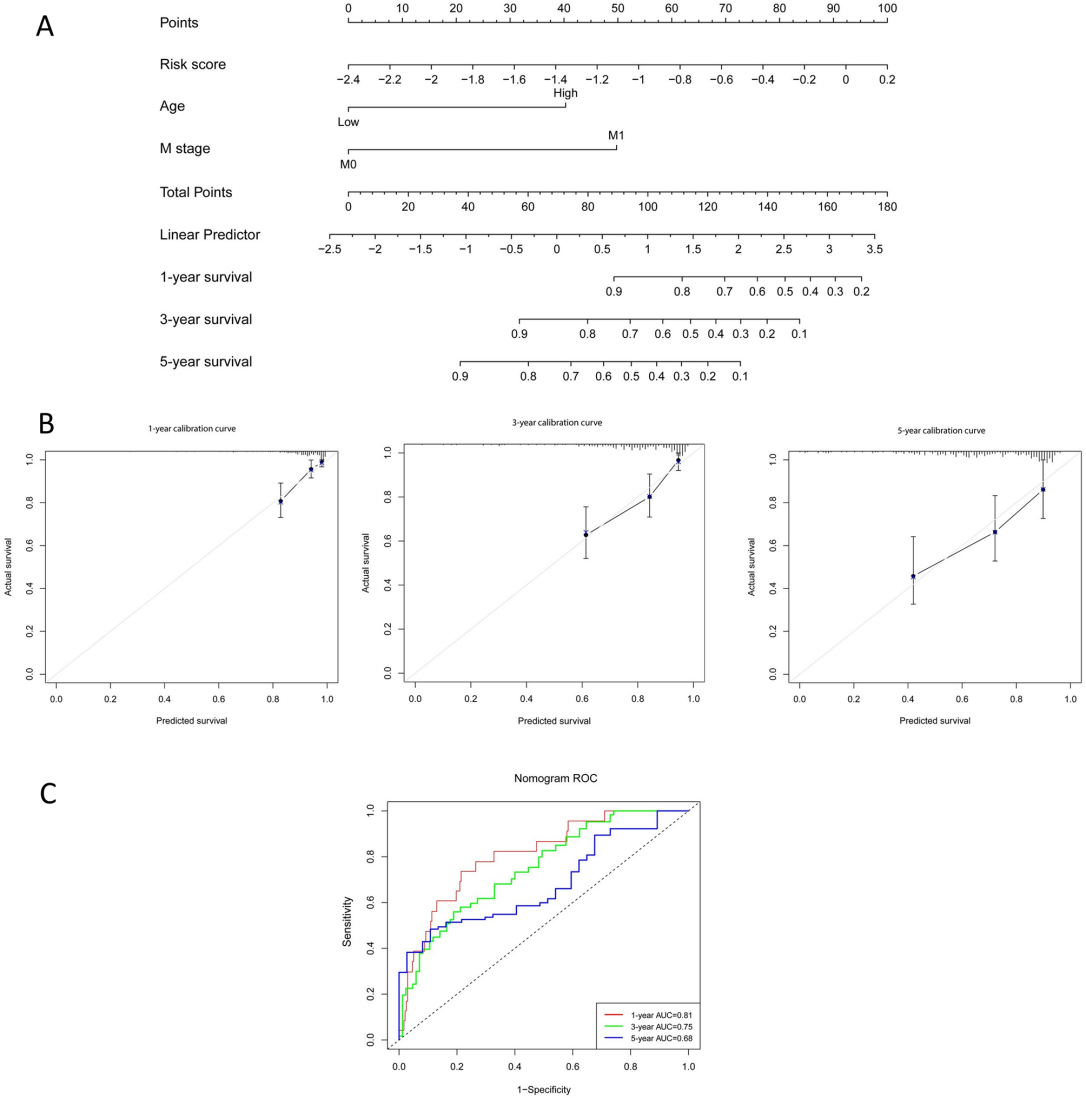
The Nomogram for predicting overall survival of CRC patients. (**A**) The Nomogram integrating the signature risk score with the pathologic characteristics for predicting OS. (**B**) The calibration curve for the Nomogram in TCGA cohort. (**C**) Time dependent ROC curve analysis of the Nomogram in TCGA cohort.

To better predict the prognosis of CRC patients, we constructed a Nomogram combining the risk score, age and M stage (Fig. 6A). Calibration curves showed that for the TCGA-CRC cohort, actual and predicted survival matched very well (Fig. 6B). The C-index of the Nomogram was 0.77 (95% CI 0.71–0.83). The AUC of the 1-, 3- and 5-year overall survival predictions for the Nomogram were 0.81, 0.75, and 0.68, respectively (Fig. 6C).

### Relationship between mitophagy signature and immune cell infiltration

**Figure 7:**
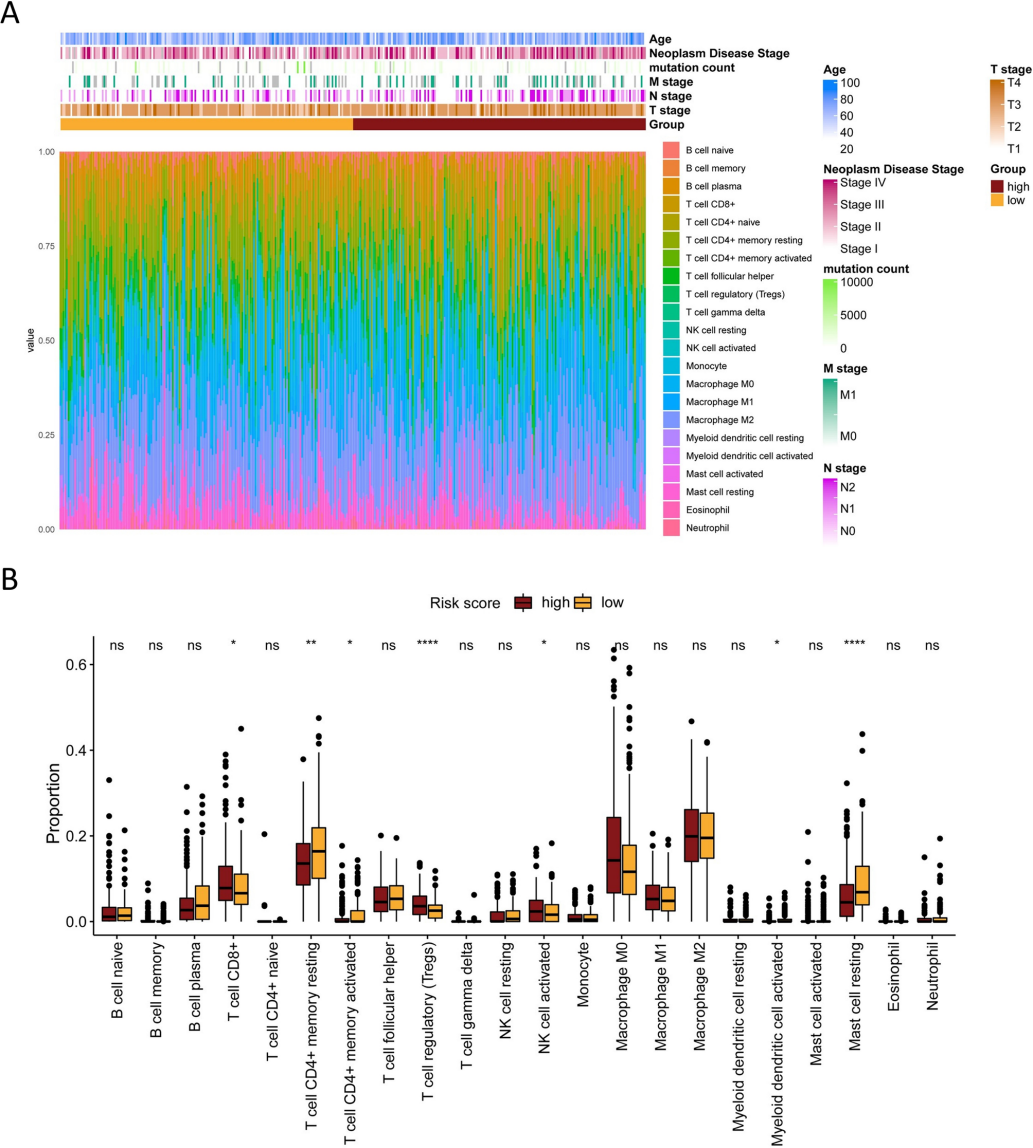
The association of the prognostic signature and immune cell infiltration. (**A**) The distribution of 22 immune cells in each TCGA-CRC patient. (**B**) Boxplots showed the differences in immune cell infiltrations between high-risk and low-risk groups.

To further explore the relationship between mitophagy-related gene signatures and antitumor immunity in CRC patients, we used the CIBERSORT algorithm to identify the immune cell infiltration of patients in TCGA-CRC. The proportion of each typical immune cell in the two risk groups is shown in Fig. 7A. Subsequently, we further compared the infiltrating abundance of immune cells in the high- and low-risk groups to explore whether there were different immune patterns in the two risk groups. Results showed that the high-risk group had significantly more enriched CD8+ T cells, regulatory T cells, and activated NK cells, while the low-risk group had significantly more resting memory CD4+ T cells and resting mast cells instead (Fig. 7B).

### Somatic mutation analysis of high- and low-risk groups

**Figure 8:**
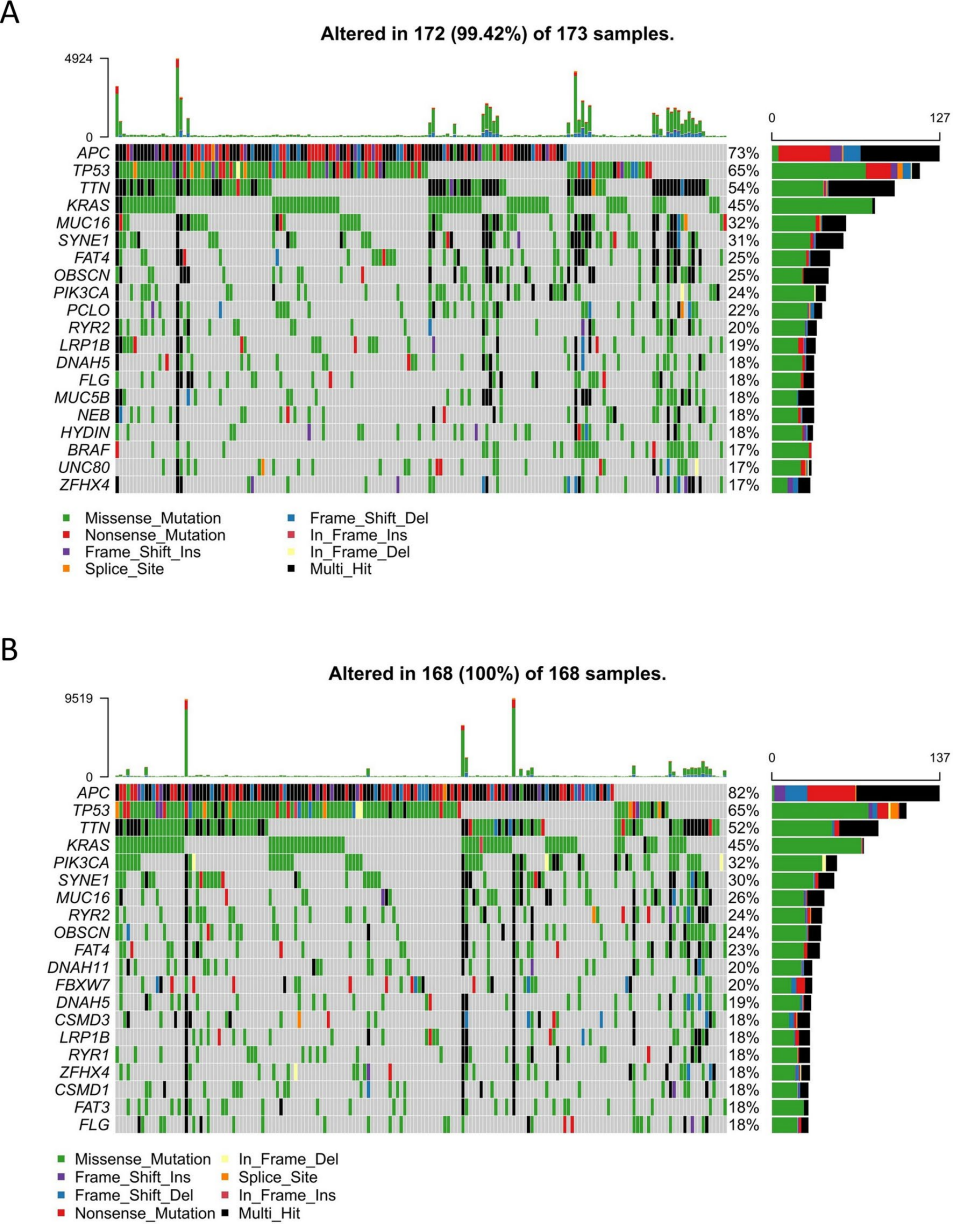
Mutation landscape of the prognostic signature in TCGA cohort. Waterfall plots of frequently mutated genes in (**A**) high-risk and (**B**) low-risk groups.

By analyzing somatic mutation data from TCGA-CRC patients, we explored the differences in genomic alterations between high-risk and low-risk groups. The oncoprint map showed the top 20 genes with the highest prevalence in high-risk and low-risk groups, respectively (Fig. 8A,B). Missense mutations were the most common mutation type in both groups. Mutation frequency of *APC*, *TP53*, *TTN*, *KRAS*, *MUC16* and *SYNE1* were all over 30% in both the two groups.

### Prediction of sensitivity to chemotherapy drugs and immune checkpoint blockade (ICB)

**Figure 9:**
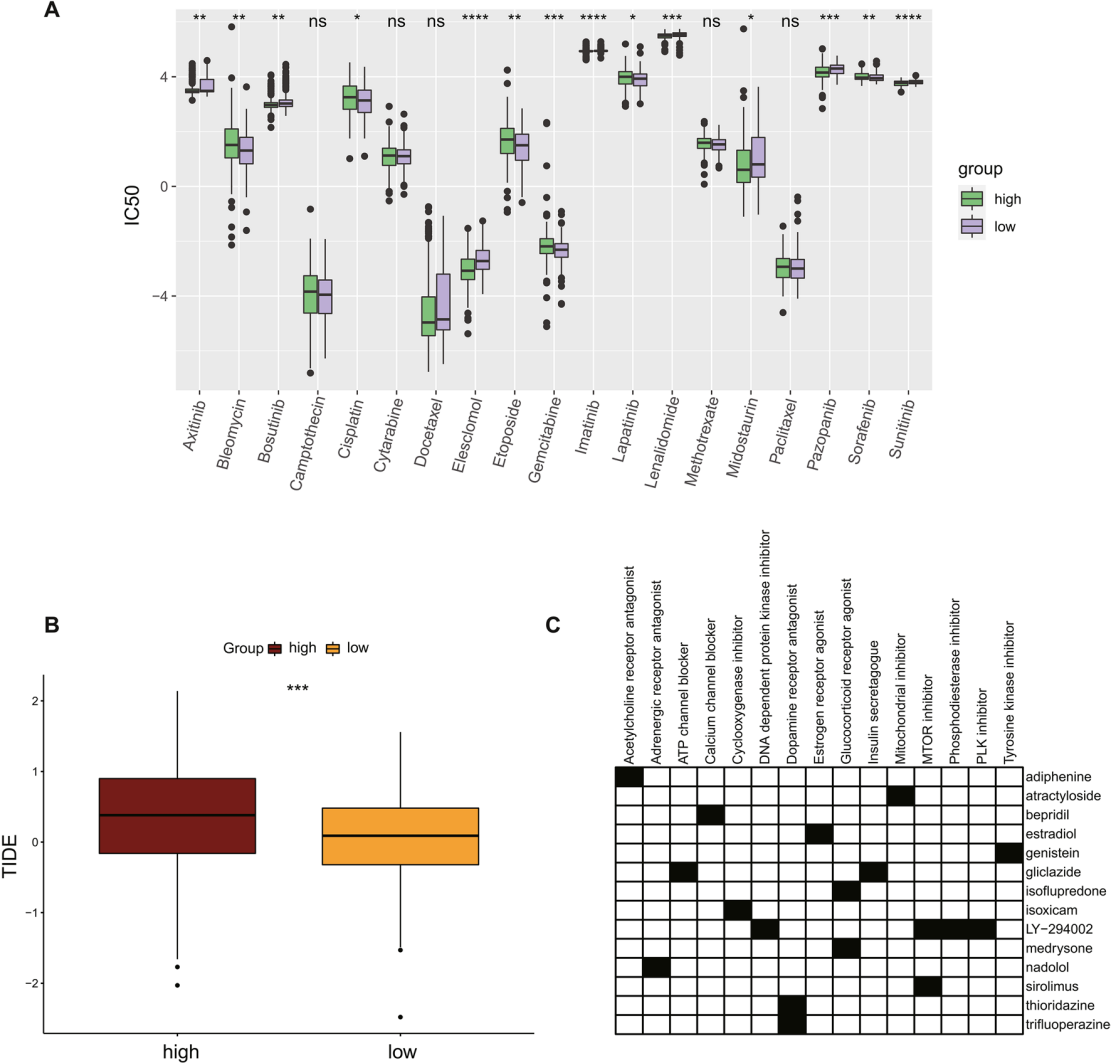
Differences in response to chemotherapy and immunotherapy among high- and low-risk group. (**A**) Boxplots describe the differences in IC_50_ levels of common chemotherapeutic agents or targeted agents between high-risk and low-risk groups. (**B**) The boxplot showed the differences of TIDE scores between the two groups. (**C**) Heatmap showed small-molecule compounds and their drug mechanisms of action.

We compared the differences in the IC_50_ levels of chemotherapy drugs or targeted drugs such as cisplatin, gemcitabine, sorafenib, and camptothecin in the high-risk group and the low-risk group. Data showed that the IC_50_ levels of bleomycin, cisplatin, etoposide, gemcitabine and sorafenib in the high-risk group were significantly higher than those in the low-risk group (Fig. 9A, p < 0.05). On the contrary, there were significantly lower IC_50_ levels of bosutinib, elesclomol, lenalidomide, midostaurin, pazopanib, sunitinib in the high-risk group compared with the low-risk group (Fig. 9A, p < 0.05), indicating that the high-risk group was more sensitive to these drugs. In addition, the TIDE score of high-risk patients was higher than that of low-risk patients (Fig. 9B).

In summary, the high-risk group is more sensitive to bosutinib, elesclomol, lenalidomide, midostaurin, pazopanib, sunitinib, while the low-risk group is more likely to benefit from immunotherapy. In order to explore potential small-molecule drugs with inhibitory effects on colorectal cancer and promote the development of new drugs, we used the Connectivity Map (CMap) database to analyze the DEGs (divided into upregulated and downregulated groups) between the high-risk and low-risk groups. CMap is a gene expression profiling database^24^. It is a biological application database established by an expert team based on the differentially expressed genes of human cells treated with small molecule drugs to reveal the correlation between small molecule drugs, gene expression and diseases. Finally, 14 small molecule drugs with anti-CRC progression effects were screened, including adiphenine, atractyloside, bepridil, estradiol, genistein, gliclazide, isoflupredone, isoxicam, LY-294002, medrysone, nadolol, sirolimus, thioridazine, and trifluoperazine, involving 15 effects mechanism (Fig. 9C).

### In vitro validation of the oncogenic function of ATG14 in CRC cells

**Figure 10:**
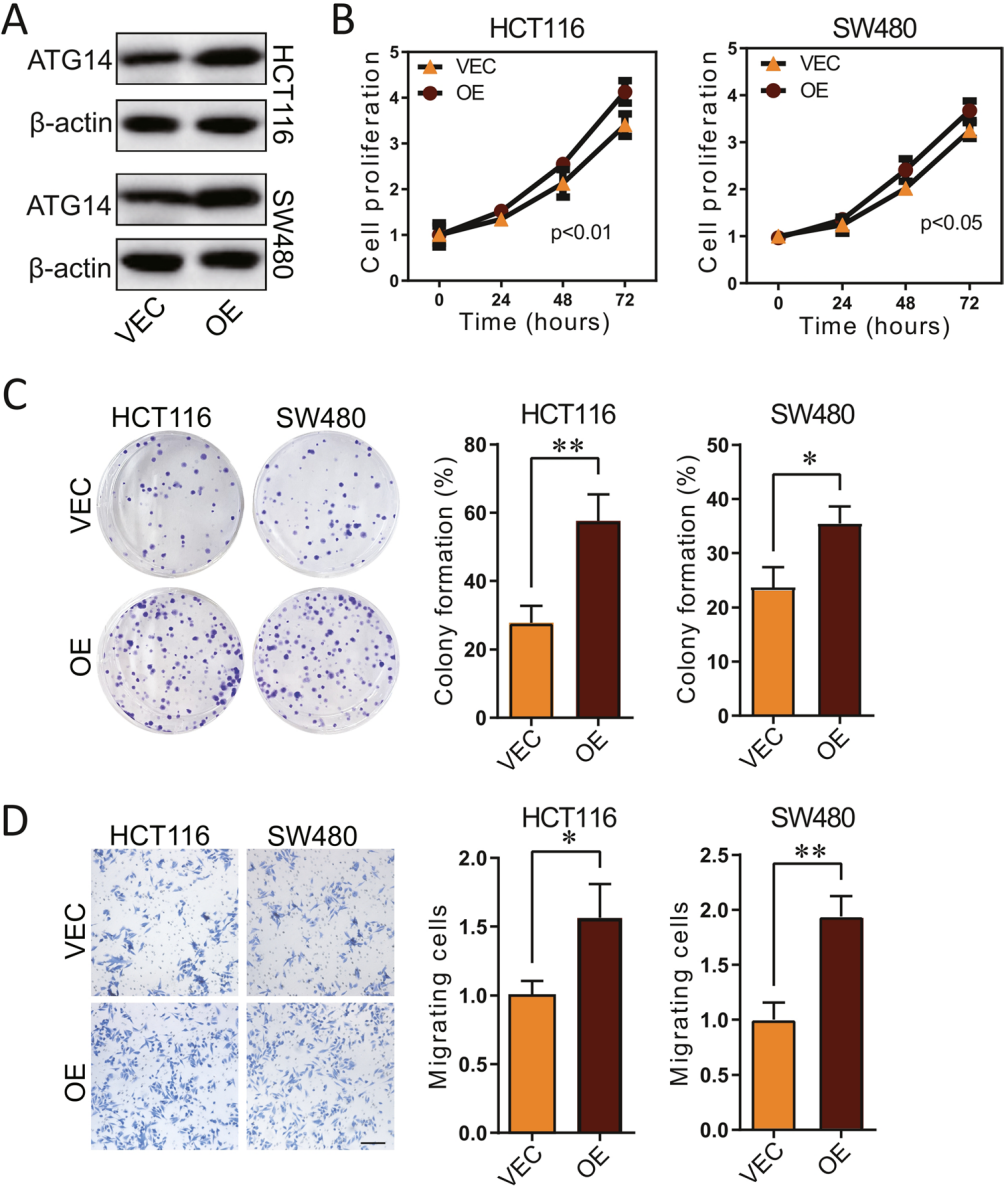
Validation of the function of ATG14 in CRC cell lines. (**A**) Western blotting of ATG14 and beta-actin in vector or ATG14 overexpressed HCT116 and SW480 cells; (**B**) Cell proliferation of vector or ATG14 overexpressed HCT116 and SW480. (**C**) Colony formation of vector or ATG14 overexpressed HCT116 and SW480. (**D**) Cell migration of vector or ATG14 overexpressed HCT116 and SW480, determined by Transwell assay, scale bar, 100 μm. *p < 0.05; **p < 0.01.

As no prior study has elucidated the precise function of ATG14 in CRC, we performed in vitro investigation to confirm if it is an oncogene in CRC as suggested by silicon analysis. By using the pCMV plasmid plasmid, we successfully introduced CRC cell lines with ATG overexpression (Fig. 10A). In both HCT116 and SW480 cells, ATG14 overexpression significantly promoted cell proliferation than those control cells (Fig. 10B). Meanwhile, we tested the effect of ATG14 overexpression on the colony formation ability in HCT116 and SW480 cells, and found that upregulation of ATG14 significantly induced CRC colony formation (Fig. 10C). Furthermore, ATG14 overexpression also promoted CRC migration which was revealed by the transwell migration assay in Fig. 10D.

## Discussion

CRC is one of the most prevalent malignant tumors in the world, and its high recurrence and metastasis rates, but low early detection rate and limited therapy options, have garnered great interest^25,26^. Mitophagy-related genes are a promising therapeutic target and prognostic indicator for colorectal cancer revealed by the growing evidence that mitophagy is inherently related to the progression and therapy of CRC^27–29^. However, the comprehensive involvement of mitophagy-related genes in the prognosis of CRC remains poorly understood.

In this study, we analyzed mitophagy related genes and identified 22 genes associated with CRC survival. Through LASSO regression analysis, 10 mitophagy related genes (AMBRA1, ATG14, MAP1LC3A, MAP1LC3B, OPTN, VDAC1, ATG5, CSNK2A2, MFN1, TOMM22) were screened out. Previous evidence has supported that some of these mitophagy-related genes involving in the development of CRC. For example, AMBRA1 is a key regulator of autophagy and apoptosis in CRC cells, maintaining the balance between autophagy and apoptosis by interacting with Beclin1^30^. Du et al. found that *OPTN ca*n be used to predict the prognosis of CRC patients^31^. Except that, VDAC1 mRNA and protein were significantly upregulated in CRC, and inhibition of VDAC1/AMPK/mTOR pathway could significantly inhibit the proliferation, metastasis and invasion of CRC cells^32^. ATG5 depletion can inhibit or promote CRC tumor growth^33^. A recent study suggested that ATG5 acts as a tumor promoter in CRC metastasis and drug resistance^34^. CSNK2A2 suppresses apoptosis in CRC by desensitizing cells to TRAIL in a caspase-dependent manner but NF-κβ independent^35^. ATG14, MAP1LC3A and MAP1LC3B have also been confirmed to be abnormally expressed in CRC and affect the development of CRC tumors^36–38^. However, there is no previous study investigating the role of MFN1 and TOMM22 in the progression of CRC, we are the first study revealing the tumor suppressor role of these two genes, Especially TOMM22 which was overexpressed in the tumor samples. Enrichment analysis of DEGs also showed that these genes were enriched in “ECM structural constituent” and “ECM receptor interaction” pathways. Studies have shown that the abnormal expression of ECM protein is associated with the carcinogenesis and poor prognosis of CRC^39,40^.

The prognostic signature constructed based on the expression of the 10 genes above showed accurate and robust prognostic prediction capacity in TCGA-CRC and external independent GEO-CRC cohort. Patients with high-risk scores had significantly worse outcomes. When patients were stratified by traditional risk features such as age, TNM stage and MSI, mitophagy-related signature still retained its predictive ability to distinguish high-risk patients. Compared with the traditional risk features of age and M stage, the mitophagy-related signature was more accurate in predicting the prognosis of CRC patients (Supplementary Fig. 2). In addition, we generated a Nomogram to quantify the risk assessment and survival probability based on risk score, age and M stage. Compared to the above factors, the Nomogram exhibited the highest accuracy and discrimination in survival prediction.

CRC patients will finally develop resistance to chemotherapy^41^, thus novel therapeutic strategies are needed. Our analysis showed that high risk patients were more sensitive to bosutinib, elesclomol, lenalidomide, midostaurin, pazopanib, and sunitinib. Among them, bosutinib (SKI-606) could reduce the growth and motility of CRC cells by preventing pp60(c-Src)-dependent β-catenin tyrosine phosphorylation and its nuclear signaling, and may be a promising choice for the treating CRC^42^. Additional in vitro studies have shown that elesclomol-induced copper chelation inhibits CRC by targeting ATP7A and modulating ferroptosis^43^. Lenalidomide induces tumor vessel normalization and improves the therapeutic index of chemotherapy in metastatic CRC in *vivo*, but further studies are needed to explore the synergistic effect between lenalidomide and conventional therapy to treat solid tumors that may benefit from tumor vasculature normalization^44^. A recent animal experiment showed that PAZ inhibited the growth of CRC, and inhibited lymph node metastasis and lymph angiogenesis in an orthotopic colon cancer model in nude mice^45^. Additionally, although sunitinib has little benefit in patients with solid tumors including CRC, it has a synergistic inhibitory effect on CRC cell proliferation when sunitinib is combined with BBSKE, which may be a potential CRC treatment strategy^46^.

Mismatch repair-deficient/microsatellite instable (dMMR/MSI) CRC tumors are highly infiltrated by immune cells^47^, and the effectiveness of ICB in dMMR/MSI mCRC has been widely demonstrated^48–51^, but ICB has shown poor efficacy in pMMR/MSS CRC^52^. PMMR/MSS CRC patients present with distinct immune profiles, giving evidence of different immune escape mechanisms, which can be overcome by individualized immunotherapy^53^. The composition of the tumor microenvironment has been shown to influence ICB responses^54^. ICB works by reinvigorating an effective anti-tumor immune response by using immune cell infiltration (primarily T cells) within the tumor^54^. The degree of immune cell infiltration plays a crucial role in the prognosis of CRC patients^55,56^. A recent study showed that resting CD4 memory T cells were the protective factor of CRC and could be used as an independent prognostic factor^57^. In various cancers, resting CD4 memory T cells were associated with increased overall survival, so the frequency of resting CD4 memory T cells predicted better survival^58–60^. And the level of mast cell infiltration in CRC is positively correlated with good prognosis^61^. In our study, the infiltrating abundance of resting CD4 memory T cells and resting mast cells was significantly higher in the low-risk group than in the high-risk group. This further confirms the accuracy of CD4 memory T cells and mast cells as predictors of CRC prognosis. Additionally, our high-risk group had higher levels of regulatory T (Treg) cell infiltration compared to the low-risk group, suggesting that Treg cells may contribute to CRC progression. Some previous studies have found that tumor Treg cell infiltration cannot predict the prognosis of CRC^62,63^. However, increased Treg cell density was associated with poor tumor differentiation and increased lymph node involvement^62^. Treg cells comprise a heterogeneous subset, some of which contribute to the progression of CRC, such as CD8^+^ Treg cells, RORγt^+^ Treg cells and IL-17-producing Treg cells^64–66^.

Different numbers, phenotypes, and localization of tumor-infiltrating lymphocytes are not only key regulators of disease progression, but also potential biomarkers for predicting immunotherapy response^67^. This shows the potential of our signature in predicting tumor immune microenvironment of CRC, which might benefit the immunotherapy. TIDE computational method, which integrates the expression signatures of T cell dysfunction and T cell exclusion to simulate tumor immune escape, can predict clinical response to ICB based on pre-treatment tumor profiles^23^. We then used the TIDE method to predict the response of high-risk score and low-risk score CRC patients to ICB therapy. TIDE score was significantly higher in high-risk group, suggesting that low-risk patients were more sensitive to ICB therapy. Thus, our signature had the potency for assisting oncologists to decide which patients are likely to respond to ICB in order to take the best course of treatment.

In conclusion, we constructed a signature that could predict the prognosis of CRC patients based on mitophagy related genes and could be used as an independent prognostic factor. The signature could also reflect the immune status of CRC patients to a certain extent. Our findings suggest that the prognostic signature may be useful for personalized treatment in clinical settings.

## Supplementary Information

Supplementary Figures.

Supplementary Table 1.

Supplementary Information.

## Data Availability

The data that support the findings of this study are available from the corresponding author upon reasonable request.
