# Supplementary material for: A mitophagy-related gene signature associated with prognosis and immune microenvironment in colorectal cancer
Source: Sci Rep. 2022 Nov 4;12:18688. doi: 10.1038/s41598-022-23463-8 (PMC9636133; doi:10.1038/s41598-022-23463-8)
Supplement: Supplementary file 1 — Supplementary Figures. [file 41598_2022_23463_MOESM1_ESM.docx]

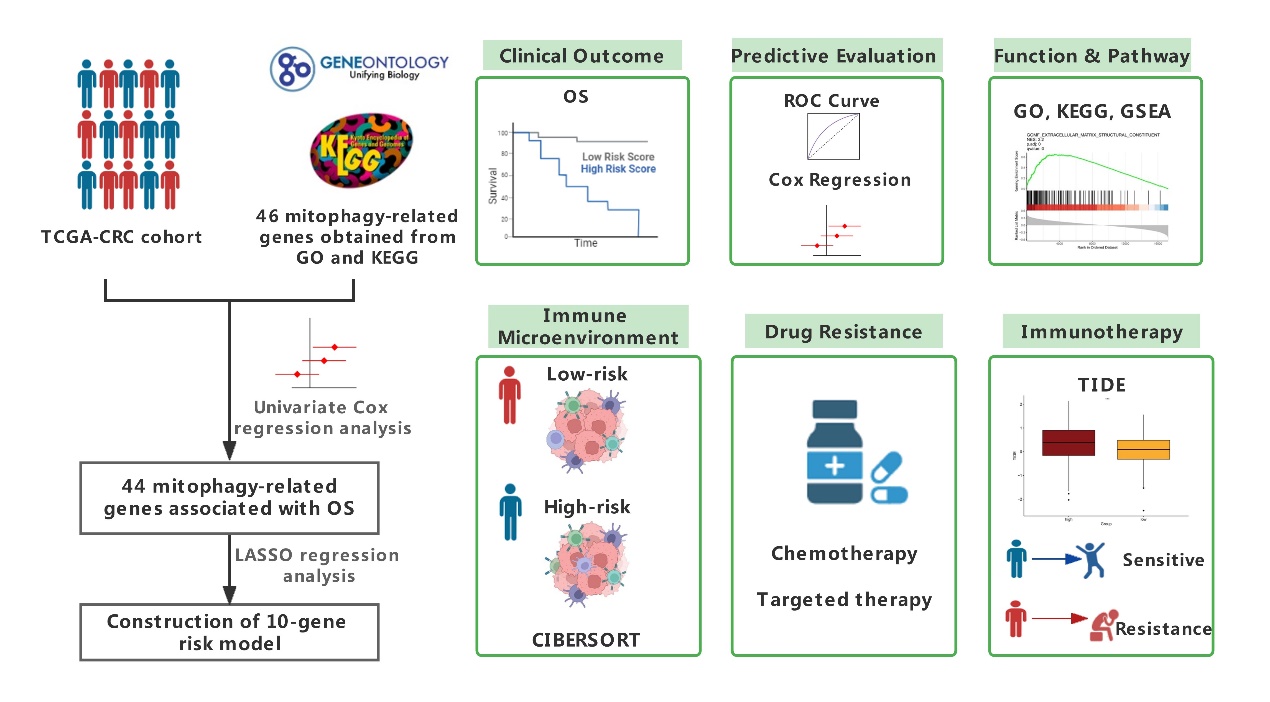


**Supplementary Figure 1** Schematic illustration of the study’s design.


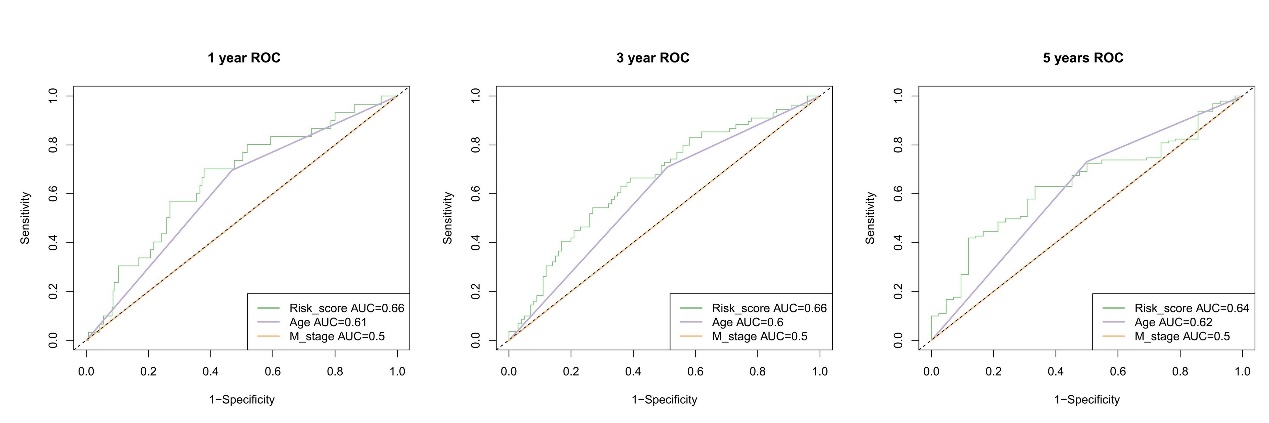


**Supplementary Figure 2** Time dependent ROC curve analysis of the risk score and traditional risk features in TCGA cohort.
