# Supplementary material for: A mitophagy-related gene signature associated with prognosis and immune microenvironment in colorectal cancer
Source: Sci Rep. 2022 Nov 4;12:18688. doi: 10.1038/s41598-022-23463-8 (PMC9636133; doi:10.1038/s41598-022-23463-8)
Supplement: Supplementary file 2 — Supplementary Table 1. [file 41598_2022_23463_MOESM2_ESM.docx]

Supplementary Table 1 Clinical characteristics of TCGA-CRC cohort.

| **Variables** | **Group** | **Total (n=354)** |
| --- | --- | --- |
| Age | ≤60 | 136 |
|  | >60 | 218 |
| Gender | Female | 161 |
|  | Male | 193 |
| Survival Status | Dead | 80 |
|  | Alive | 274 |
| Stage | Ⅰ | 54 |
|  | Ⅱ | 128 |
|  | Ⅲ | 110 |
|  | Ⅳ | 51 |
| T (Tumor) | T1 | 9 |
|  | T2 | 55 |
|  | T3 | 244 |
|  | T4 | 46 |
| N (Lymph Node) | N0 | 194 |
|  | N1 | 96 |
|  | N2 | 63 |
|  | NX | 1 |
| M (Metastasis) | M0 | 245 |
|  | M1 | 48 |
|  | MX | 58 |
