# Supplementary figures and images for: A mitophagy-related gene signature associated with prognosis and immune microenvironment in colorectal cancer
Source: Sci Rep. 2022 Nov 4;12:18688. doi: 10.1038/s41598-022-23463-8 (PMC9636133; doi:10.1038/s41598-022-23463-8)

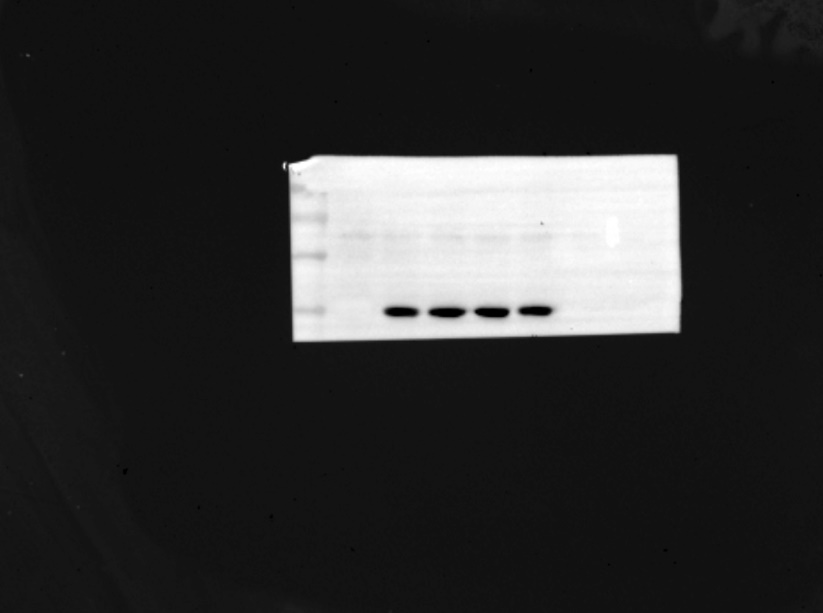


β-actin


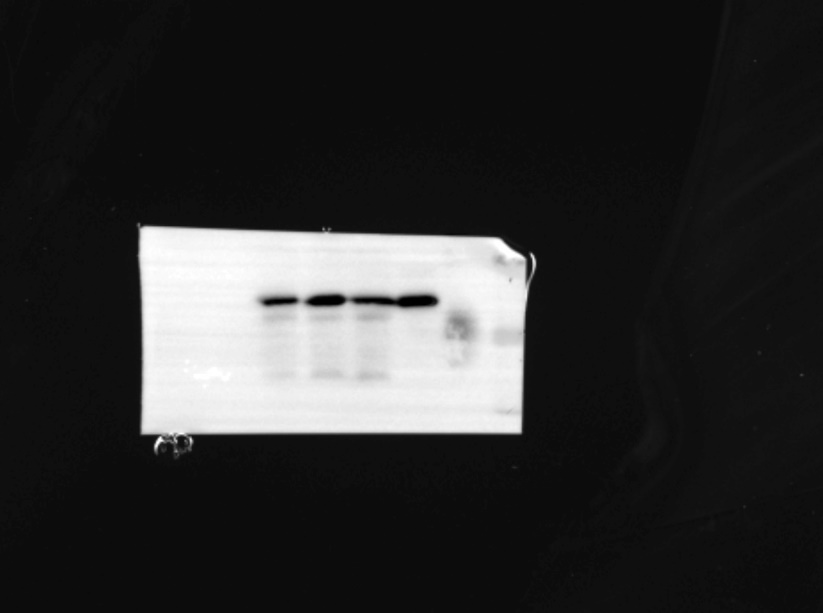


ATG14

Supplement: Supplementary file 3 — Supplementary Information. [file 41598_2022_23463_MOESM3_ESM.docx]
